# Atmospheres, landscapes and nature: Off-road runners’ experiences of well-being

**DOI:** 10.1177/1363459318785675

**Published:** 2019-02-20

**Authors:** Sara MacBride-Stewart

**Affiliations:** Cardiff University, UK

**Keywords:** environment and health, phenomenological approaches, theory

## Abstract

This article reflects on the relations between health and natural landscapes. The study explores how the landscape context – its textual and sensory aesthetics – positively shapes experiences and perceptions of the landscape, for those people who seek out natural environments for health. While health promotion is designated along the lines of encouraging choice or improving access to natural environments, this article wants to show how physical activities are intertwined with atmospheres and affects emanating from the natural and human world. An in-depth case-study of trail running across two sites (New Zealand, United Kingdom) is used to analyse the interconnections between health landscapes. It finds that when participants say that landscape ‘matters’ for health, they are referring to: (1) aesthetics and feelings, (2) flexibility and adaptiveness and (3) exploration and adventure. Avoiding the conclusion that the landscape is merely a resource for health, the analysis confirms that it is the complex of spaces, social practices, along with their physical fleshy selves, minds and emotions, and the particular quality of the earth beneath them, that gives rise to positively perceived health, for both immediate and enduring benefit.

## Introduction

The accepted claim – that natural environments are good for human health (see MacBride-Stewart et al., 2016) – must be re-examined in light of the rapid pace at which social and physical environments are changing (Nisbet et al., 2011). A decrease in the availability of biodiverse environments, along with increases in urbanisation, changes in land use, and the development of peri-urban spaces is argued to have resulted in a disconnection from nature, with negative consequences for human health (Nisbet and Zelenski, 2011). At the same time, the value of this connection between natural spaces and people, for health, is increasing in research and policy circles. Philosophers (Böhme, 1993; Menatti and Casado da Rocha, 2016), geographers (Richardson and Mitchell, 2010; Van den Berg et al., 2014) and health researchers (De Vries et al., 2003; Hartig, 2008; Nutsford et al., 2013) have sought to describe and provide evidence of the effectiveness and conceptual importance of the natural environment for health. Despite an interest in the connections between humans, landscape and health, this body of work lacks substantial evidence, and a comprehensive theory, or guidance for planning (Menatti and Casado da Rocha, 2016).

This article attempts to respond to this question about the (dis)connection with nature. It considers how the ‘special’ qualities or complementary role of aesthetics and landscape shape meanings of health. It attempts to do this using the concept of ‘atmospheres’, described later (Böhme, 1993). The paper begins by outlining the key challenges in rethinking health as emotively and materially linked to the environment. Consideration is given to the connections that occur at the level of surface, terrain and underfoot. The interest here is in what is felt and perceived, as part of the ambition of people to respond actively and physically to the changing world around them. Bringing these ideas to their conclusion, the objective of the study is to explore how the landscape context – its textual and sensory aesthetics – positively shapes experiences and perceptions of health (Gobster et al., 2007).

There are risks in this approach. The passions, desires and agential dimensions of health align with contemporary theories and health definitions that support ideas about human autonomy and self-management (see World Health Organization [WHO], 1948 definition). However these approaches tend to ignore the ways in which health choices and actions are structured, regulated and constrained. To address this issue, Menatti and Casado da Rocha (2016) note that a landscape approach in which the structural, as well as cultural and physical, dimensions of place and space are acknowledged, provides the missing link for advancing our understanding about why some groups of people appear to seek ‘contact with nature’ for health and well-being. Contact with nature refers to being-in-nature, and importantly, it does not assume that ‘use’ is synonymous with the connection to nature. While public health treats landscape as a ‘black box’ according to Spaargaren (1997), other disciplines aligned to geography have always regarded the idea of ‘landscape’ as a conceptually important spatial unit. Here, natural and social systems interact, and landscape is the product of intersecting various social practices (Smaldone et al., 2005). The paper uses Böhme’s (1993) idea of atmosphere to help us think about how recognisably rich or unique landscapes, which fall under the category of protected landscapes, render allegedly material structures accessible to affective and spatial analysis.

This article seeks to contribute to an understanding of how the relationship between landscape aesthetics and positive pro-health behaviours, might go some way to protecting and preserving these landscapes for future generations (and minimise the impact of overdevelopment or over use; Gobster et al., 2007; Nisbet et al., 2011). The project might also contribute more widely to work on restorative (Ulrich, 1983) and/or therapeutic environments (Gesler, 2009). The goal is to recognise how human feelings, values, attitudes and identities are geographically embedded, and engaged in shaping landscapes for health (Maller et al., 2009).

## Health, physical activity and greenspace

In recent decades, our understanding of how the experience of physical activity in nature might promote health has been advanced through studies on aesthetics, motivations, and affective benefits of activities in urban and rural greenspaces, parks and wilderness (Ulrich, 1983). Two distinct themes of work on health, physical activity and greenspace have emerged, which are briefly summarised here. The first part reviews the literature on medical evidence and social determinants that seeks to connect landscapes to the salutogenic or health-promoting benefits of exercise (Antonovsky, 1996). The second part reviews the literature on ‘restoration’ that connects philosophical and psychological theories of place focusing on stress reduction and attachment to place (Hartig, 2008; Van den Berg et al., 2014).

Research has been particularly interested to show whether physical activity in outdoor space is associated with improvements in physical and psychological health. The salutogenic factors are summed up by Mitchell and Popham (2008: 1655) who propose that ‘green spaces independently promote physical activity’, or more generally that natural environments ‘encourage healthy behaviours’. The majority of these studies have used quantitative measures to compare an activity in one type of environment to another. A number of studies suggest those who perceive their living environment to be green (Kemperman and Timmermans, 2014) and those who live in greener areas (McMorris et al., 2015) had higher levels of physical activity than those who did not (Kemperman and Timmermans, 2014; Paquet et al., 2013; Tamosiunas et al., 2014). However, others have found no association between exposure to greenspace and levels of physical activity (Ord et al., 2013; Tamosiunas et al., 2014; Triguero-Mas et al., 2015). The disadvantage of a ‘greenness comparison’ is that it does not provide more than the descriptive qualities or scale of the greenspace. Social factors are important as these effects decrease with age (McMorris et al., 2015), the toxicity or pollution of the area in which you exercise (Sharman et al., 2004; Wong et al., 2007), and gender (MacBride-Stewart et al., 2016).

In a medical context, questions have been asked about the specific health benefits of participating in outdoor activities. Reduced risks like cardiovascular disease and obesity, and a longer life expectancy (McNiel et al., 2012; Richardson and Mitchell, 2010), improvements in depression and quality of life (McNiel et al., 2012) and reduced scores for mental health risk (Mitchell, 2013) have been reported when comparing people who regularly exercise in natural environments to people who do not use these environments, and for people who live in closer proximity to greenspace (Bixby et al., 2015; De Vries et al., 2003; Nutsford et al., 2013; Tamosiunas et al., 2014; Triguero-Mas et al., 2015). Certain health conditions like attention-deficit hyperactivity disorder (ADHD) and depression, also benefit therapeutically from greenspace (Faber Taylor and Kuo, 2009). However, this literature does not confirm whether it is availability, or attractiveness of greenspace, that facilitates positive mental and physical health (Hartig, 2008).

The literature on restorative effects seeks to understand how positive affects can be achieved through having access to a pleasant environment (Maller et al., 2009). The criteria for ideal restorative environments described in the literature are that they are biodiverse, they contribute to a sense of being away, they support a large range of activities and thus are compatible with users’ expectations (Hartig, 2008; Van den Berg et al., 2014). Large-scale areas of nature, for example, may be more important for staying healthy because they are argued to provide a greater opportunity for reflection and a deeper level of restoration (Van den Berg et al., 2014). Similarly, Hunziker et al. (2007) add that more time in a place increases attachment to it. Along the same lines, Hartig (2008) argues that when experiencing nature, individuals feel a distance from the demands of everyday life along with the possibility for ‘aesthetic appreciation’. These are qualities that built environments supposedly do not possess, and therefore, visits to a natural environment are arguably better than visiting a built-up environment (De Vries et al., 2003; Van den Berg et al., 2014).

However, it is also possible that feeling about a place can be negative or ambivalent (Relph, 1976). The capacity of a nature space to be ‘restorative’ also differs by gender, with women experiencing very dense or wilderness spaces as stressful, or unsafe and unsuitable (Van den Berg et al., 2014). Certainly, the need for restoration and the capacity of the environment to provide that – that is, whether it is attractive enough – are also important factors that shape the experience of place (Hartig, 2008; Van den Berg et al., 2014).

Sociological work on embodiment has identified how the strands of research identified here are deeply embedded in dualisms that separate physical from psychological processes, humans from nature. Thus, written into the very argument about greenspace and health is a form of analysis that produces an abstract and universal account of health and place, unobservant of the peculiarities of context and human difference (Gabrielson and Parady, 2010). Authors who analyse the materiality of this environment, have largely circumnavigated the health dimension of this relationship (Alaimo, 2010; Fox and Alldred, 2016; Grosz, 1998). What is commonly over-looked in the sociological and public health fields are the non-instrumental interactions, and the emotional and aesthetic relationships between people, and the natural environment, and health. While these relationships have been somewhat addressed by Tuan (1977) - who defined a person’s affective attachment to place as topophilia (Menatti and Casado da Rocha, 2016) - overwhelmingly, aesthetic experiences often appear in the academic literature as representations or symbolic images of nature that are passively taken up by humans for their health (Summers et al., 2012). There is a gap in understanding the active appropriation and engagement with the physical landscape for health. To some extent, this is why a different way of thinking about human–landscape interactions in public health is needed. It is possible in this context to raise questions that ask what are natural landscapes for, what do they do, and how do they fit into the broader need to be actively and agentically involved in health and physical activity (see Xu and Fox, 2014, for a broader discussion).

## Off-road running

In Spaargaren’s (1997) view, the question of ‘what people do in natural environments’ helps us address the question of ‘what these spaces do for people’. In this study, off-road running helps us to understand how greenspaces might be integrated into people’s everyday lives, rituals and practices. It is helpful to explore how the experience of running off-road is mediated and felt, and how individuals themselves define and prioritise their personal health and well-being in relation to natural spaces.

Recreational running is by far one of the most common activities that people do after walking (Qviström, 2016). As a popular physical fitness/health practice, running has become an obvious target for public health (Hitchings and Latham, 2016). Off-road running is good exemplar of a physical activity that exploits ‘nature trails’ (Qviström, 2016). Off-road running in particular fits with level of moderate to strenuous activity preferred by people who visit a National Park.^1^

Off-road running is associated with idealised images of going ‘back to the nature’ and encounters with ‘untouched landscapes’ (Qviström, 2016). It involves the selection of many landscape features – terrain, scenery – opposite to traditional urban practices of track and road running, and their associated surfaces. Off-road running is usually represented as a physically skilful practice that centres on expertise about how to move through the landscape. In general, it has comparatively few formal rules and resources, but its shared ideologies regarding respect for the landscape, the environment and other runners, informs the practice of running materially as well as socially.

Only a few studies explore relationships between runners and landscape in depth (Bale, 2004; Latham, 2015; Qviström, 2016). Authors such as Latham (2015) link growing numbers of runners to the increasing stresses of modern, urban life. In this context, off-road running has been represented as a strategy for managing the pressures of urban life and the unnaturalness of built environments (Latham, 2015). An increase in the number of people using the environment for physical activity and recreation is a significant concern to off-road runners (Hitchings and Latham, 2016). However, this ‘back-to-nature approach’ is being challenged by the appearance of more formal events, organised by companies who often operate as part of global franchises. As off-road running constitutes an important ‘transactional zone’ where the public health and economic imperatives for the production of fit, active bodies coalesce with the desirable natural environment and the individual desire to be healthy, it proposes to be an interesting practice to study.

## Atmosphere: the embeddedness of affective practices in natural environments

This section considers how Böhme’s (1993) concept of atmosphere – which can be understood as the ‘affective mood which spatial arrangements stir in the sensual bodies of their users’ (Reckwitz, 2012: 255) – can contribute to the question of what it means to be in nature, and what natural landscapes do for people and their health. This links to the definition of landscape as an ‘aesthetic experience’ which Gobster et al. (2007: 964) define as ‘a feeling of pleasure attributable to directly perceivable characteristics of spatially and/or temporally arrayed landscape patterns’.

Böhme (1993) begins with the assertion that the concept of atmosphere can be useful where there is an intention to express an indeterminate nature, making it relevant when discussing the indirect nature of the health benefits of natural landscapes. He argues that at the same time, atmosphere appears to have a deliberately evocative emphasis – communicating something that is beyond clear or discernible rational explanation, but which is assumed when we speak of a ‘good’ atmosphere or the ‘serene atmosphere’ of a scenic landscape. As such, the concept of atmosphere in practice appears to emerge between a deliberate indeterminism and clear affective expression. This enfolding, he notes, has ontological implications:

we are not sure whether we should attribute them [atmospheres] from the objects or environments from which they proceed, or to the subjects who experience them. We are also unsure where they are. They seem to fill the space with a certain tone of feeling like a haze. (Böhme, 1993: 114)

In his account of atmosphere, Böhme (1993) notes how attempts to describe what landscape is and what it does have often been limited to symbolic and representational accounts of feelings that tell us little about the sensation of actively *being* in a place. This has led to interest in how natural environments might produce evidence from ‘the ground up’; and a focus on what is experienced sensorially. What Böhme’s approach (1993: 116) makes clear is that a ‘good atmosphere’ or a ‘positive health outcome’ ‘cannot be grasped solely through its concrete qualities’. Notably, the perceiver moves in the landscape – giving the landscape and the people within it an agentic and a relational quality (Menatti and Casado da Rocha, 2016). Menatti and Casado da Rocha (2016) add that meanings derived from the environment occur across many levels from what is underfoot, to surfaces, substances, and events relevant to a person’s life. In viewing human responses to the landscape as active, ‘individual preferences, choices, and actions … aggregated over broader social and societal levels, have the potential to change landscapes, regions, ecosystems, and other environmental phenomena’ (Menatti and Casado da Rocha, 2016: 965).

At its most basic level, this takes into account the connection between landscape and health that emerges precisely because humans are perceiving and sensing bodies (Menatti and Casado da Rocha, 2016). Atmosphere-at-a-distance is also possible. This is confirmed by research that shows the positive effects on health when images of nature are projected into a classroom (see also Benfield et al., 2015). In this example, atmosphere-at-a-distance narrows the experience by portraying a singular visual or aesthetic element. Briefly, the spatial and scalar dimensions of atmosphere touched on here highlight the importance of landscapes within the direct experience of individuals, and the multiple intersecting aesthetic and affective experiences evoked through engaging in landscape. Furthermore, by paying attention to the agency of objects and places, as well as people (Frohlich and Abel, 2014), this approach highlights individual and collective forms of doing, performing and feeling, in response to natural, ecological features of the landscape (Menatti and Casado da Rocha, 2016). Consequently, physical activities in outdoor spaces are not only rational health practices, they are also a means to activate the material and aesthetic, sensations and perceptions, between people and environments (Reckwitz, 2012).

## Methods

The study evaluates the experiences of off-road runners registered at a running event in the United Kingdom and New Zealand. The UK event was a locally organised women-only event with marathon and relay distances, while the NZ event was one race in an off-road running series, for adults and children,^2^ with a maximum distance of 21 km. This research uses a case-study approach because it allowed for the detailed examination of off-road running in two locations (Abercrombie et al., 1984). By selecting unique events, the research avoided the tendency common to some qualitative research methods, to generalise experiences. Rather, the selection of cases was underpinned by the recognition that diverse context-dependent, as well as practical and accessible, data have the potential to yield rich information about how and why people use natural environments (Flyvbjerg, 2006).

Event selection was opportunistic, with the researcher contacting the organisers of the women-only UK event because it was promoted as a local, as well as a nationally relevant, celebration of women’s inclusion in long-distance running. The NZ event was used as a ‘comparator’ for the UK event as both were held in a protected area: a Regional Park area of native bush and an Area of Outsanding Natural Beauty (AONB)/World Heritage Site, respectively. The events differed in terms of (1) gender balance (single-sex vs mixed), (2) location (UK/NZ) and (3) amount of time spent running off-road, for example, UK participants spent more time off-road. However, events were similar in terms of demographics based on (1) equal age distributions, (2) similar patterns of average weekly running distance and (3) balancing factors, for example, a greater proportion of UK participants who had been running over 10 years matched the greater proportion of NZ participants who had run more than 10 marathons.

The research data were collected over two research ‘event’ days. Each research event had three main elements: (1) online survey, (2) vox-pop interviews and (3) mobile, ‘go-along’ interviews (Carpiano, 2009; Evans and Jones, 2011). The online Qualtric-hosted survey had 28 (UK) and 34 (NZ) qualitative and quantitative questions on off-road running benefits, barriers and costs. The survey was promoted to competitors in pre-race and post-race information. There were 260 responses (140 UK, 120 NZ) to the online survey. The survey reveals important information about well-being and recreational disturbance, while the NZ version survey included additional questions about biosecurity measures in place during the event. Seaton (1997) observed that while surveys are classic tools for use in event-based research, they often lack important information on the organisational, contextual and experiential aspects of individual events. They advocate supplementing data with additional methods.

In the ‘go-along’, the interviews were conducted while a researcher ran the race route. Mobile interviews are particularly well suited to capturing new experiences, and observations of embodied, ‘in-the-minute’, contextual encounters (Carpiano, 2009; Evans and Jones, 2011). As only a handful of participants could be interviewed like this, a set of ‘vox-pop’ media-style interviews was also conducted (Richardson et al., 2014). ‘Vox pop’ are ‘short, street’ interviews suitable for ‘one-time, short-run, events with fast exit rates’ (Seaton, 1997: 25). This method was designed for this study to capitalise on the opportunities to talk to runners before and after their event (Dowling et al., 2016). The emphasis was on the immediacy, the specificity and the peculiarity of the off-road event experience (Brace and Geoghegan, 2011). For the vox-pop, a team of researchers (4 UK, 2 NZ event) interviewed individuals or groups of runners and families gathered at the start and end of the race. People were randomly selected although an effort was made to select for diversity (i.e. age, ability). There were four brief questions related to enjoyment, health benefits and value, and sustainability of off-road running for the vox-pop and go-along interviews. Interviews lasted between 5 and 30 minutes, with most lasting more than 10 minutes. All were digitally recorded and transcribed. In total, there were 132 interviews (82 UK, 50 NZ of which 36 were group interviews).

The key challenges of mobile interviews are impacts of weather, time of day and noise (crowds, wind, event announcements; Carpiano, 2009). Good media-quality recording equipment was required. In this research, many of the methodological challenges were also opportunities for rich data – as the method allowed the participants to ‘feel the terrain’ and the weather. The multiple voices and an outdoor soundscape gave the interviews a dynamic quality (Garcia et al., 2012). As participants are able to set the pace of the interview, some interviews were brief but interviews generally produced focused, rich data with interviewers taking opportunities to encourage participants to be reflexive and to expand on ‘common sense’ responses (see Hollway and Jefferson, 2000). Mobile interviews generate analytic challenges as participants’ commentary often needs to be linked to landscape aspects (Carpiano, 2009). Challenges were addressed by researchers making a verbal note of landscape features at the time of the interviews, and having a clear structured set of topics to cover.

The qualitative data from the survey and interviews only is presented here. The analytic process is drawn from Hollway and Jefferson’s (2000) psychosocial approach. The data was reviewed independently by two researchers who identified key themes. The initial conclusion was that these themes could be descriptively organised around (1) the event (environmental mitigation, reasons for taking part, atmosphere) and (2) impacts of long-distance, off-road running (well-being, recreational disturbance). They constituted mostly a ‘common-sense’ analysis that needed a critical, theoretical lens in order to address the problems outlined in the literature review and specifically to enable attention to context. The answer was found in the theory of atmosphere in which the crucial motivation for investment in particular discourses was the relationship between landscape and health. Using this particular theory of human health–landscape relationships enabled the author to focus in depth on the key areas of (1) social practices, (2) spaces, (3) perceptions and (4) moods that constitute off-road running as an affective and relational space. The themes were verified by returning to the literature on affective landscapes (Mitchell, 2003; Newman et al., 2017). At this stage, the framework of (1) aesthetics and feeling, (2) flexibility and adaptiveness, and (3) exploration and adventure was applied to the analysis.

## Analysis

The next section is organised around the main analytic themes, which include extracts from the interviews.^3^ The participants’ accounts of the main benefits of exercising off-road preface the analysis. These benefits were described as the ‘enjoyment of the natural environment’ along with a ‘sense of positive feeling’, ‘getting fitter and stronger’. The former was expressed as the opportunity to feel a part of the environment, resonating with so-called ‘wilderness philosophy’; that exercising in more remote areas away from people provides the opportunity for immersion in the beauty and serenity of nature, detaches from focus on pace adding both challenge and interest, and the opportunity to explore and discover new places or trails. Further explanation was provided by the participants in the interviews and open-text responses. These data are expanded in the next sections.

### Aesthetics and feeling (sensations and affects)

The runners in this study used a range of feelings to describe this experience of nature – from uplifting, beautiful and awesome, to a sense of being transported – while running off-road. Sometimes, it was enough for the runners to identify this sense of beauty but to leave its unspecified quality unexplained. Examples of extracts from which this analysis was derived are presented below, and to emphasise the discursive nature of these responses, more than one extract is sometimes included:

It’s beautiful the scenery … And to be able to run somewhere like that is absolutely amazing. In those moments we forget what we were doing … wow look how high up I am. (UK3P17)

We always call it tonic for the soul. Always. Feel like a different person when you get back from running in the woods. (UK3P27)

This understanding of nature as producing a positive affect for running is consistent with a hedonic approach, which defines well-being in terms of pleasure and happiness (Ryan and Deci, 2001; Summers et al., 2012), as well as feeling better. This pleasure was expressed as the (unspecified) enjoyment of countryside, as the atmosphere of being surrounded by or immersed in nature. Often hedonistic pleasures triggered things that were physically sensational, like the fitness or play:

It’s like the sense of doing something I wouldn’t normally, wading through a mud puddle like a kid, is quite fun. It’s about pleasure. Fitness.

From a sensory perspective, nature was represented as sights (scenery, variation of plants/green, lichen), smells, sounds (or the absence of them) and touch (on ground). The capacity to have ones senses engaged created an affective (dis)connection that was generally associated with improved mental health, as described below:

It’s fresher air … in the woods. It’s just fresher and cleaner … It just clears the head.

Calmer …

And you notice the seasons. I ran with the bluebells for about a month this year. Ran in the snow

In the woods when it’s raining, you hear all the leaves. (NZ2P08Group)

A particular quality of the landscapes referenced in the experience of off-road running is its peaceful, quiet or unspoilt nature, producing a sense of well-being. This peace and calmness included having a quiet atmosphere unspoilt traffic or by other people, including family and kids. This thinking atmosphere was argued to constitute a ‘headspace’ in which one might ‘forget the world around’ and, ‘lose oneself’. Some runners referenced this to the idea of runners ‘flow’ – a cultural reference to a desirable state that runners strive for and only rarely achieve: ‘you feel amazing … that feeling of mind and body working together’ (UK3P09).

I like how peaceful and calm it is and how it clears your head … Just to be on your own for hours. It’s nice to have complete silence. (UK1P04)

In addition, because of the runners’ close attention to what was underfoot, the qualities of the ‘terrain’ was important in terms of how it felt, its variety and the focus on the natural landscape that it required (Antonovsky, 1996; Corazon et al., 2011; Gobster et al., 2007). Notably, the NZ group referenced the physical benefits of the terrain comparatively more than the UK group, emphasising the strengthening and preserving of joints; although both discussed the need to focus on what is underfoot. This difference may have been due to difficult terrain encountered in the NZ event (including steep downhill and muddy tracks):

You have to think a little bit about where you’re putting your feet. There’s a lot more to look at so you don’t really think, I don’t personally think about anything except the run. (UK3P18)

Conversely, environments that were perceived as degraded, unattractive or unnatural were unlikely to produce an atmosphere of positive well-being (Ryan and Deci, 2001):

I think the human impact is the most [un-aesthetic] thing to see in a forest, you’re walking through a pristine forest and you see a cut down tree or rubbish, it just spoils the experience a little bit. (NZ2P26)

To briefly sum up, the sense of well-being generated by off-road running was characterised as a set qualities or positive *atmospheres* associated with nature, which produced a sense of well-being. The qualities most commonly referred to were (1) spatial dimensions of openness and space (associated with scenery and view), (2) quality of peace and calmness, (3) quality of beauty or aestheticism and (4) qualities of the terrain. Notably, these qualities appear to be distinguishable and coherent, and combine physical and affective elements. They also appear to act together in a relational way making it difficult to assign any more or less significance to each quality. Well-being, for example, was attributable to a sense of freedom (of unlimited scale) as well as an atmosphere of absence, tranquillity and the sensory absorption of local natural features.

### Flexibility and adaptiveness: (complex of social practices)

In both contexts, differences between the off-road and road running were often directly compared. Comparisons were made between the qualities of space (freedom vs restriction), terrain (mud vs tarmac), beauty (architecture vs scenic landscapes) and peace (traffic vs birdsong). Road running for example is conducted on hard pavements, with traffic, noise and pollution. In addition, road running reflects norms for exercise that are commodified and demand a fast pace of life. These ideas develop the perception that the city environment is an exhausting and unlikeable place to exercise, and that mental health benefits particularly are more limited:

On road you’ve got the fumes of the cars. (UK3P17)

Road running is monotonous. You’re a machine, you just turn off and run. (NZ1P06)

Off-road running was presented as a contrast to modern life. Modern life was frequently represented as busy, frantic, but it also has the atmosphere of being urban, competitive, costly and driven by technology. Running off-road is constituted an escape from technology, from pressures and demands of others and of work. It was frequently presented as being ‘good for your feelings, you feel better after running’ (NZP21). In particular, the use of natural environments for exercise was presented as the pursuit of a different kind of energy, a detachment, and less mentally tiring space. Most often, the contrast to modern life and an ambivalence with traditional norms of road running was presented as a combination. Here, the spatial and affective qualities linked to produce an atmosphere of well-being. The opportunity for this escape was regarded as necessary to maintain physical and mental state of health. Many talked about using running to not only manage pressures of work but also depression and anxiety:

I like the feeling afterwards, can just get out and run in the countryside. It’s beautiful. You can clear your head. Had a stressful day at work with your family [you] can just go out and forget about everything. I like off-road. It’s more of a challenge. (UK2P02)

The participants commented on a range of relations to modern life that provided insights into the ways in which running was an escape from but also a realisation and a fulfilment of the expectations of modern life. Off-road trails reflect variety and interest and this help to actively sustain running but also offer a means for fully participating and managing the demands of modern life. Off-road running is described in this context as: ‘a break from everyday life … a reward’, as ‘little adventures … to drag me away from work and my house’. Technological metaphors used by participants emphasise this relation to modern life:

You have a computer and you defrag it don’t you? … It puts everything in the compartments. Tidies it all up. Then off you go and you work better. I find running does that for me. By the time you’ve finished, your head is completely clear of all that fuzz and dirt. Gets rid of it and you feel great. (UK3P03)

The physical challenge of trails themselves is presented as an ideal metaphor for modern life. The ability to be flexible, to be self-managing, to seek out challenge, to exercise autonomy, and to be independent, coheres not only with ideals about the modern worker and desirable modernity (Sointu, 2005) but they also reflect ideals about how individual and communities should participate in health within a neoliberal context of health (Clarke and Shim, 2011; Rose, 2007). While academics have reported on how modernity demands flexibility and responsiveness of individuals, runners describe similar reasons for and effects of off-road running (expressed below):

The scenery. The freedom. The challenge …You’re having to pit yourself against it. In life actually there’s ups and downs – there’s stingy nettles; [laugh] there’s rocky terrain. So it’s fantastic to get out and meet the challenge. (UK2P05)

These terrain demands were physical (‘rocky’, ‘undulating’) and affective (‘pit yourself against it’). They represented elements of challenge which, when overcome, were contributors the sense of well-being achieved through running. Consequently, the findings assert that trail running – with its demands for flexibility and adaptiveness – is as much as an assertion of modern life, as the separation from it.

### Exploration and adventure: (complex of spaces)

The runners who did not live in proximity to trails noted the particular challenges that they faced in engineering an escape from modern life. The reported barriers to long-distance off-road exercise included difficulty finding routes or challenge in finding time away from family, and difficulty accessing trails due to living in a city. There were also some concerns specifically related to natural environments such as fear of cows, trips and falls, lack of daylight and personal safety concerns. For this group of runners, many of these barriers were reported as incidental, able to be overcome as part of a responsive and flexible approach to maintaining physical activity.

Notably, barriers seemed to reduce as people became more or less familiar with the physical environment and terrain. It is a finding that is also consistent with Menatti and Casado da Rocha’s (2016) notion of the terrain as active, with the potential for meanings and experiences derived from the environment to change as they aggregate. This process was described by the number of respondents who indicated that they now had few insurmountable barriers to prevent them undertaking long-distance off-road exercise, although this had not always been so. Even ideas about whether trails were unsafe could change:

Once upon a time I wouldn’t [feel safe] but now actually I would feel safer running out along this coast path than I would running in a built up area. (UK3P01)

While fear or lack of safety was a concern raised by some, getting lost was an advantage when linked to a sense of adventure and exploration commonly associated with travel in new locations:

It’s the unpredictability of being off-road. I have been lost countless times in marathons but it never bothers me … I don’t mind doing those extra miles because it’s another bit of exploration … seeing parts of the country other people don’t get to see. (UK3P01)

The research found that runners in particular regarded the natural landscape in ways that might traditionally be associated with perspectives on recreational tourism, rather than restorative environments per se. Off-road running – particularly formal events – was associated with visiting and seeing places and scenery, or areas that were new, or that one might not normally visit. Many people at the New Zealand event for example had not previously visited the local Regional Park in which the event was being run. Participants noted how the event was often organised as a special trip, including family or club members:

Anywhere I go on holiday, the first thing I do is look for a run route. I find places that other people I go on holiday with don’t find … up on a coastal area that’s perhaps a bit run down. I find areas of amazing beauty. I just love exploring- by foot. (UK3P01)

These places did not need to be far away. The adventure could be in exploring new routes, location or paths that were local. This links to the earlier ideas about the variety of terrain or scenery, as a key element of pleasure in off-road running:

For me it’s just a mini adventure. Wellbeing is just a side effect of it, I don’t explicitly go up there to be all healthy and stuff. It’s more like I need to go out, get away from the computer I guess so just a mini adventure. Get it done in the morning, middle of the afternoon I get home again then back to the routine. (NZ2P19)

This tourism is not solitary but includes groups, families that people travel with or the companionship and connections they make when they get there. Some of the participants used the event as a regular family day out, others used them to enjoy the company of friends and running partners. Overall, the participants’ accounts highlight the social opportunities of created by travelling to an event:

It’s companionship …

Encouragement. As I said there’s six other of us all similar age. [We] do different events – trail ones. It brought us together and we all encourage each other. As a wellness thing it’s companionship. Support. Fun.

We were talking in the car coming about what’s next. (NZ2P09 Group)

One of the final questions in the survey and the interviews asked participants how it might be possible to make off-road running sustainable, for individuals or for groups. It was in response to this question that similar concerns posed in the ecological management, recreational disturbance and ecotourism literatures was raised by participants. Sometimes known as the ‘parks versus people’ debate there was a concern that allowing too many people into protected areas risk damaging paths and native species (a particular concern in the New Zealand context):

It’s tough cos you wanna make people enjoy as possible, but at the same time you need to control the traffic through these areas. A balance between somehow being in control [ensuring] people don’t just go off the trails and ruin the rest of the park. Stick to the trails. Get as many people through will enjoy the benefits, so can [ ] be sustained. (NZ2P03)

While the runners felt that they were ecologically aware and responsible – picking up litter, sticking to trails more generally – mostly participants felt that off-road running had little impact on the landscape. The natural landscape appeared to sustain its role in supporting the health of individuals, and participants often failed to make the link between the local event that were participating in and benefitting from, and the wider responsibilities linked to protecting greenspace for health.

## Summary of findings

In general terms the research supports the literature, finding that natural landscapes do appear to play an important role in facilitating and promoting exercise, and in sustaining this engagement. Running on trails was regarded as good for joint health, stress reduction stress, and as an escape from everyday life. Off-road running appears propelled by a sense of the importance of landscape not found in other accounts of running (Latham, 2015). This research also confirms the claims made in the literature about off-road running: that it is a physically skilled, back-to-nature activity, that is useful for managing the pressures of modern life and the unnaturalness of built environments. More than that, runners appeared passionate about their running and its health benefits, rarely critical or scrutinising of the possible constraints for others, or its possible environmental impact.

More importantly, the study hoped to explore how the landscape context – its textual and sensory aesthetics – positively shapes experiences and perceptions of the landscape, for those people who seek out natural environments for health. In asking this question, the paper picks up on the claims by Menatti and Casado da Rocha (2016) that landscapes are a conceptually important spatial unit where desirable health practices and environments intersect. The three themes – aesthetics and feeling, exploration and adventure, flexibility and responsiveness – are all derived from paying close attention to the health–landscape relationship in the context of off-road running. Some of the detail of this relationship was provided through a focus on Böhme’s (1993) concept of atmospheres which is attentive to the affective moods produced by spatial arrangements, for example, the pleasures of being isolated or immersed in nature. In considering the affective and aesthetic relationships between people and natural environments in off-road running – the research shows an overwhelming sense of enjoyment and associated affects including positivity of mood, attention, focus, energy and so forth. Running positively for health was constituted by a sense of beauty in nature, the enjoyment of adventure, and strength and capability that came from responding to the demands of the environment.

This study does have limitations. The study was focused on obtaining environmentally contextualised perspectives of runners using the natural environment for health. The choice of methodology did not allow for a detailed analysis of the structural or institutional shaping of experiences. Some reflection on the role of instuitions and governance emerged in the context of discussions about biosecurity controls in the New Zealand context; however, this aspect was not designed as a comparative aspect. As already mentioned, some of the landscape features captured in mobile methods were only indirectly referred to and often needed interpretation by the researcher. Further research is needed to explore the limits of language for expressing nature relationships, and the broad differences in meaning between the NZ and UK context. One of the reviewers pointed out that including a women-only event had relevance for the analysis based on the literature finding that women have qualitatively different experiences of nature. Gender is not directly addressed here, but combining the data from the two distinct case studies was deliberate so that womens’ voices are not subordinated or singled out for special attention, but appear as part of the multiple voices engaged in meaning-making. This ‘radical’ approach is advocated by Hollway and Jefferson (2000) who encourage the application of emotional rather than cognitively derived research logics, in order to disrupt the rationally conceived, often male-dominated, logic of social science analyses. Following this, emphasis was given to the meanings contained within the diverse range of statements and their potential to produce deliberately confusing or contradictory findings. The challenge is to generate a theoretical rather than a methodological generalizability, which it is hoped was achieved.

## From landscape as a health resource, to practices, spaces and affects

What this research shows is that our traditional understanding of what landscapes do for people can be partially answered by what people do in landscapes. In this research, people ran – off-road. However, they rarely expressed being solely motivated by physical health goals, as is often the expectation of health policy. Rather participants acknowledged the demands of modern life and actively sought out practices, spaces and affects that enabled them to actively manage its pressures, finding ways to remain effective, productive and ‘modern’. In addition, off-road running was presented as a desirable practice that looked more like training for adventure, exploration and fun, than a traditional incitement towards health activity. Understanding health in this way provides some practical guidance for the promotion of physically active healthy lives.

With an atmosphere of positive health clearly established, this research raises the question of why this matters for studies of greenspace and health. The findings show us that these affective atmospheres of landscapes are actively desired and sought out and that landscapes are responded to and related to – not passively – but through the engagement of physical bodies with sounds, smells, touch and sights, and the physical movement of bodies to, within and away from these spaces. The ground turns ankles, undulates, throws up views at the top of a mountain and becomes mud. This physicality – and the space in which it occurs – matters for health. What emerges is a powerful healthy circuit between the embodied human with their physical fleshy self, their mind and emotions, and the particular quality of the earth beneath them and the landscape surrounding them. It is these dimensions that may help us better understand what people seek from natural environments.

It is important to sound a note of caution. Greenspaces have been shown to have an important connection to modern stressful lives. As work and economics are not distributed evenly, care needs to be taken to ensure than resources are distributed equitably, and not simply according to professed need. Furthermore, Gobster et al. (2007) remind us that while what is considered good atmosphere may become aligned with health goals, it is not necessarily aligned with good ecological quality. What is good for human health and positively attributed to humans may not be positively correlated with ecological health.

## Notes

1. In a recent survey of 1500 visitors, Brecon Beacons National Park, Wales (BBNPA, 2017) found that over 65 per cent of those surveyed preferred moderate to strenuous paced activities during their visit.

2. Children under 18 years were not interviewed as part of this study.

3. Extracts used here are direct quotes and are presented as they appear in the recordings and are consistent with a commitment to representing participants’ voices (Hollway and Jefferson, 2000). To avoid the sense that these accounts are attached to individuals, more than one extract is sometimes presented to better reflect their discursive nature.
